# Metastatic neuroendocrine tumor masquerading as liver abscesses

**DOI:** 10.1002/ccr3.3224

**Published:** 2020-08-05

**Authors:** Abhimanyu Aggarwal, Madhuvan Gupta, Joseph Gabriel, Durane Walker

**Affiliations:** ^1^ Division of Infectious Diseases Department of Medicine Baystate Medical Center‐University of Massachusetts Medical School Springfield Massachusetts USA; ^2^ Division of Pathology Hindu Rao Hospital Delhi India; ^3^ Division of Medical Oncology Baystate Medical Center‐University of Massachusetts Medical School Springfield Massachusetts USA

**Keywords:** aspiration studies, imaging studies, liver abscess, liver mass, neuroendocrine tumors

## Abstract

Fever and deranged transaminases with liver mass(es) on imaging mandates further evaluation of the mass(es) and should be followed radiologically and clinically. In the absence of a definitive diagnosis, repeat biopsy should be done.

## Graphical abstract

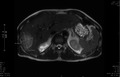

## 1 INTRODUCTION

Several intra‐abdominal pathologies from the gastrointestinal tract have a tendency to spread via the portal venous system to the liver, including infections and malignancies. In cases of acute onset fever, right upper quadrant abdominal pain, deranged liver function tests and the presence of liver masses, the diagnostic approach should start with, but not be limited to, an infectious work up. Follow‐up on clinical symptomatology and imaging can be extremely helpful in confirming the diagnosis. We report one such case of a middle‐aged adult who presented with the above‐mentioned signs and symptoms not improving on an 8‐week course of antibiotics and with patient‐physician perseverance was diagnosed with a metastatic neuroendocrine tumor.

## 2 CASE REPORT

A 52‐year‐old male with a past medical history significant for well‐controlled hypertension, psoriatic arthritis controlled on prior sulfasalazine therapy (completed 5 years prior) with no history of immunomodulators, presented to the outpatient clinic (day 1) with one week of subjective low‐grade fever and chills in the month of September in Massachusetts, US. He was very active outdoors for recreation and denied any significant travel history. Social history was negative for tobacco, alcohol, or illicit drug use. Complete blood count and comprehensive metabolic panel, including liver function tests (LFTs), were normal. On day 3, ELISA test for Lyme C6 antibody was positive and he was started pre‐emptively on oral doxycycline 100 mg twice per day.

**Figure 1:**
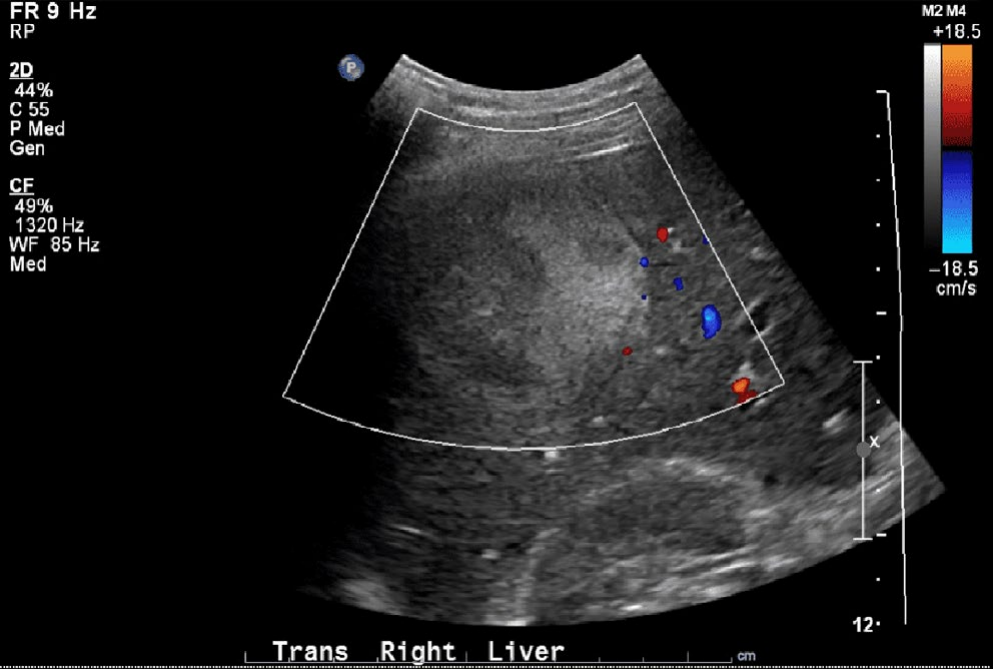
Ultrasound Doppler of the larger liver mass in segment VI measuring 5.9 × 5.3 × 5.5 cm in size and with peripheral blood flow

**Figure 2:**
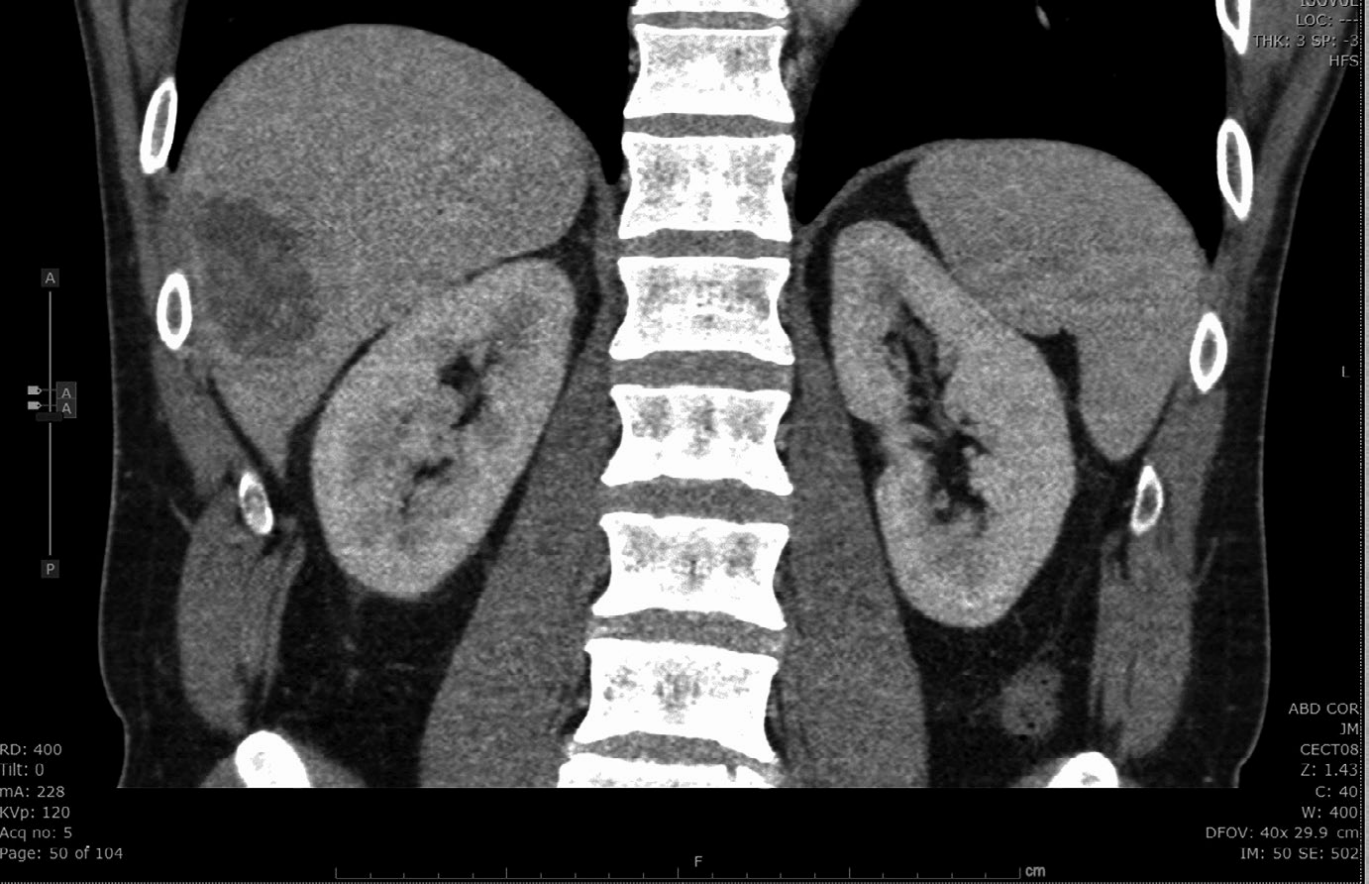
Computed tomography (CT) scan, during hospitalization, showing multiseptated complex larger mass with thick wall and internal fluid attenuation, measuring 5.5 × 3.8 × 5.2 cm in size

On day 5, the fever was persistent and he developed right upper quadrant abdominal pain. He presented to the emergency department and routine blood work now showed elevated aspartate aminotransferase (AST), alanine aminotransferase (ALT), and alkaline phosphatase (ALP) levels (Table 1). Right upper quadrant ultrasound showed two solid masses in the right hepatic lobe with heterogeneously increased parenchymal echogenicity. The larger lesion measured 5.9 × 5.3 x 5.5 cm in segment VI, and smaller one was 2.5 × 2.3 × 3.1 cm in segment VIII. There was evidence of peripheral blood flow to the masses on Doppler (Figure 1). The differentials suggested in the impression included metastases, primary liver malignancy, atypical hemangioma, and less likely abscess. Computed tomography (CT) abdomen showed the two same masses with thickened walls and internal fluid attenuation (Figure 2). There were additionally a few hypodensities in the liver that were too small to be characterized. Differentials in the impression included pyogenic liver abscess and hepatic metastases with a recommendation for tissue sampling for further characterization. The patient was subsequently admitted to the hospital.

**Table 1:** Laboratory values from different stages of presentation

	Day 1	Day 5	Day 37	Day 76	Day 191, two days post‐op (different laboratory, same units)
White blood cell count (reference range 4‐11 K/mm^3^)	9.7	8.7	4.9	9.3	16.4 (reference range, 4.5‐11)
AST (reference range, 0‐38 units/L)	32	70	23	50	404 (reference range, 10‐40)
ALT (reference range, 0‐41 units/L)	36	113	20	79	345 (reference range, 10‐55)
ALP (reference range, 40‐119 units/L)	98	158	72	142	75 (reference range, 45‐115)
Total bilirubin (reference range 0‐1.2 mg/dL)	0.4	0.2	0.3	0.4	1.2 (reference range, 0‐1.0)

**Figure 3:**
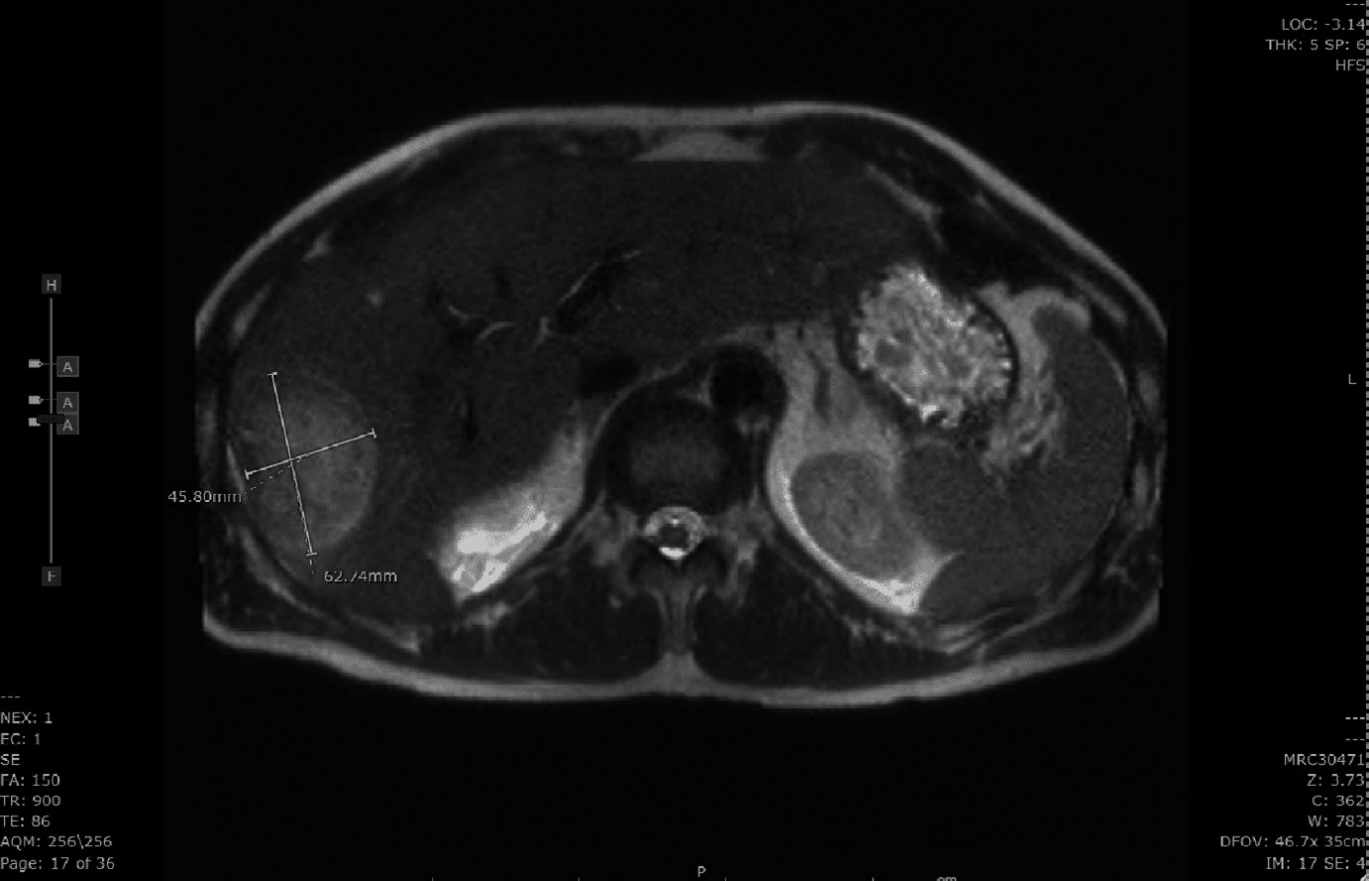
Magnetic resonance imaging (MRI) scan, during hospitalization, showing heterogeneous mildly T2 hyperintense and T2 hypointense lesion within the right hepatic lobe measuring 4.6 × 6.3 × 6.0 cm (transverse by AP by craniocaudal length), with a continuous thick irregular wall and internal solid and cystic components, with vascular enhancement of the wall and internal solid components

Infectious diseases consultation was requested for antibiotic recommendations. Western blot for Lyme disease showed 5 positive IgG bands (bands p93, p41, p39, p23, and p18) and no positive IgM band. Doxycycline was discontinued. CT‐guided aspiration of the larger lesion was done on day 6, and only a small amount of bloody aspirate was obtained. Aspirate cultures were negative for growth but gram stain showed 2 + polymorphonuclear leukocytes and no organisms. Cytology was negative for malignant cells but showed reactive ductal epithelial groups and scattered acute inflammatory cells. Blood cultures were also negative. Entamoeba serology was negative and viral hepatitis (A, B & C) serology as well as PCR for Anaplasma, Ehrlichia, and Babesia were also negative. Following the aspiration, intravenous (IV) piperacillin‐tazobactam was started. Magnetic resonance imaging (MRI) of abdomen with contrast done the same evening described the same lesions. The larger lesion had continuous thick irregular wall and internal solid and cystic components with vascular enhancement of the wall and internal solid components (Figure 3). Transient hypervascular enhancement to the adjacent parenchyma was seen on arterial phase. The smaller lesion was only mildly heterogenous and showed restricted diffusion and moderate arterial enhancement greater than parenchyma eventually becoming hypoenhancing relative to the liver in later phase. Also noted were multiple subcentimeter T2 hyperintense and T1 hypointense lesions without arterial enhancement that represented cysts. The report was described as most consistent with abscesses and much less likely malignancy based on the appearance. The patient improved clinically as did his LFTs while on piperacillin‐tazobactam, so it was recommended that he complete a 6‐week antibiotic course with follow‐up imaging prior to stopping. He was also evaluated by the gastroenterology team simultaneously who agreed with our assessment of presentation to be consistent with an infectious process, especially in view of an unremarkable esophagogastroduodenoscopy and colonoscopy within the prior two years, with the former being done because of a family history of Barrett's esophagus.

On day 30, he had a follow‐up CT abdomen showing the persistent 2 lesions with the smaller lesion being of same size but the larger lesion decreasing in size down to 4.5 cm, unclear whether it was an effect of aspiration or antibiotics. The radiologist's impression suggested follow‐up imaging to exclude the possibility of malignancy. On day 36, he followed up with infectious diseases physician and given only very minimal improvement on repeat imaging, the concern for an alternative diagnosis was raised. He completed the 6‐week course of IV piperacillin‐tazobactam followed by two additional weeks of oral amoxicillin‐clavulanic acid. On day 47, he followed up with gastroenterology and agreed with the recommendation of completing the antibiotic course of 8 weeks for likely liver abscess.

On day 75, he called our ID office with concerns of feeling feverish again. On day 76, blood cultures, complete blood count, and comprehensive metabolic panel were drawn and he was found to have deranged AST and ALT again (Table 1). Additional tests with the relapse of symptoms included normal serum prostate‐specific antigen level, normal serum alpha‐fetoprotein level, negative repeat Entamoeba serology, and negative Echinococcus serology. On day 82, MRI abdomen with contrast was repeated that showed mild decrease in size of the larger lesion and evidence of increased signal suggesting interval hemorrhage within the lesion but otherwise continuous thick irregular walls and internal solid and cystic components with vascular enhancement of the wall and internal solid components. The smaller lesion increased in size to 5.4 × 3.4 × 5 cm in size with no changes in signal and enhancement characteristics. Satellite lesions also did not change during this time. Radiology impression was that the decrease in size of the larger lesion argued strongly against a neoplastic etiology; however, repeat aspiration and attempt at core biopsies of both the lesions were recommended for confirmation. Antibiotics were deferred at this time since the patient was otherwise hemodynamically stable and also to increase the diagnostic yield.

On day 104, ultrasound‐guided biopsy and aspiration of the larger liver mass were done. Two core biopsies and 2 fine‐needle aspirates were taken from the mass. Aspirate and biopsy cultures were again negative for any growth, including fungal and mycobacterial cultures. Aspirate cytology showed rare atypical cells present in the background of extensive necrosis. Biopsy pathology showed evidence of well‐differentiated grade 2 neuroendocrine (carcinoid) tumor with uninvolved adjacent liver, immunohistochemical staining was positive for synaptophysin and chromogranin and a Ki‐67 labeling index of 16.8%. On day 113, he was seen by the local oncology team as an outpatient. On day 133, positron emission tomography (PET) CT GA‐68 dotatate study showed abnormal focal uptake in the distal small bowel near the caecum with metastatic lesions seen throughout the liver consistent with prior CT. On day 189, with surgical oncology consultation at a higher institution, he underwent right colectomy, right hepatectomy, partial left hepatectomy, with surgical pathology revealing grade 1 carcinoid of terminal ileum with 4 of 43 lymph nodes positive and 17 right liver and 3 left liver metastases resected.

One month following the surgery, he reached out to us to thank our team for our persistence in obtaining a correct diagnosis and subsequently connecting him with the appropriate specialists. He is recovering well and with no evidence of residual disease is currently not on any chemotherapy or hormonal therapy.

## 3 DISCUSSION

The classic approach to a patient presenting with fever and right upper quadrant abdominal pain traditionally starts with thorough history and physical examination, followed by pertinent laboratories including liver function tests. Interpreting the results of these liver function tests can be quite stylistic with the differential diagnoses differing by age group, clinical symptomatology, geographical location or local endemic diseases, travel history, occupation, hepatocellular versus cholestatic pattern of derangement as well as degree of AST/ALT elevation. The most common cause of asymptomatic elevation of transaminases is nonalcoholic steatohepatitis worldwide, while symptomatic elevation of transaminases is most likely caused by viral hepatitis. Travel history can appropriately raise the suspicion for amoebic liver abscess or parasitic infestations like liver fluke. Direct liver damage versus biliary obstruction is reflected in elevated alkaline phosphatase and gamma‐glutamyltransferase levels in the latter. More than 10‐fold elevation of AST/ALT is usually seen in ischemic or drug‐induced liver injuries.^1^

With appropriate narrowing of the differential diagnoses, the next step involves imaging studies. Ultrasound, computed tomography, and magnetic resonance imaging all have their benefits in localizing the site of abnormality. For the purpose of restricting the discussion to our case, we will focus on the findings of single or multiple liver masses which can be infectious or malignant in origin. Infectious causes include pyogenic liver abscess, amoebic liver abscess, mycobacterial liver abscess, or parasitic cysts/abscesses. Malignant causes include primary liver malignancy or liver metastasis from distant sites including colorectal cancers, ovarian cancer, prostate cancer, breast cancer, etc Rare pathologies like hepatic adenomas or hemangiomas can also present as such. Imaging findings, regardless of the modality, can be nonspecific with radiologic impression still giving rise to wide differential. Large abscesses appear as hypoechoic or hyperechoic masses depending on internal echoes due to septa and debris on ultrasound.^2^ Infectious masses can have almost the same appearance as malignant liver metastatic lesions, especially if the latter has internal necrotic debris. Contrast‐enhanced CT findings may demonstrate low‐attenuation masses with an enhancing peripheral rim, which are grossly no different in appearance from metastatic liver lesions from a malignancy,^3^ unless certain spectral CT quantitative parameters are measured.^4^ MRI with contrast in the dynamic phase usually shows central low T1 weighted and high T2‐weighted signal intensity but depending on the protein content, internal signal intensities may vary. The outer layer can be hyperintense as well as perilesional edema may appear as high signal intensity on T2‐weighted images.^2^, ^3^ PET/CT scans may offer some advantage with hyperactivity in neoplasms^5^ provided there are no necrotic areas in the core.

In early 20th Century, the most common cause of hepatic abscesses was pylephlebitis secondary to appendicitis. Over the course of time, biliary tract disease has become the most frequent culprit.^6^ Simultaneously, mortality from hepatic abscesses reached 75%‐80% in the early 1900s but with improvement in diagnostic and treatment modalities has fallen to 10%‐40%.^7^ One of the most common predisposing factors for hepatic abscesses is diabetes mellitus with several pathophysiological features contributing to the high risk including altered neutrophil metabolism.^8^ Also the increasing use of proton pump inhibitors has been associated with an increased risk of hepatic abscess formation presumably because of increased gastric pH due to these medications which decreases the natural gastric defense against bacteria and hence translocation via the portal system to the liver.^9^ Interestingly, nonmetastatic colorectal cancers have also been associated with formation of hepatic abscess, presumably due to local destruction of the mucosal barrier and hence, bacterial translocation and invasion.^8^ *Escherichia coli* was the most common pathogen isolated worldwide from hepatic abscesses prior to the 1980s, with the trend shifting to *Klebsiella pneumonia* in more recent years.^10^ Malignant abscesses can be due to secondary infection of a primary liver tumor, metastatic liver lesion or superinfection of spontaneous necrosis. Common reported presentation includes fever, abdominal pain, and hypotension.^11^

For the accurate diagnosis of a liver mass, fine‐needle aspiration (FNA) is considered the gold standard ^12^ with a yield that is highly sensitive.^13^, ^14^ The presence of liver lesions raises the suspicion for infections or malignancy; however, the process of fine‐needle aspiration and its interpretation is fraught with pitfalls.^15^ A pyogenic liver abscess is characterized by the presence of neutrophils, multiple microbes as well as necrotic tissue on Gram stain.^16^ Microbes may not be identified depending on specimen handling as well as pretreatment with antibiotics prior to aspiration. Similarly, the core of malignant lesions can also undergo necrosis and can be characterized by the presence of necrotic debris along with neutrophils.^17^ One study reported superiority of needle biopsy over FNA cytology, by the demonstration of better architectural, cellular as well as immunohistochemical evaluation of liver lesions in biopsy specimens.^18^

Several case reports have been cited in the literature where the diagnostic dilemma just based on radiological findings were clarified further by histopathology studies. In one case report,^19^ multi‐dimensional CT was suggestive of metastatic liver lesions until it was confirmed as pyogenic liver abscesses by appearance of ultrasound‐guided aspirate of the largest lesion and its microbiology results. Similar case reports where lesions earlier thought to be malignant or metastatic lesions eventually diagnosed as pyogenic,^20^ tubercular,^21^ syphilitic,^22^ toxocariasis,^23^ amebic,^24^ and actinomycosis ^25^ liver masses have been reported. Other case reports have cited initial infectious‐appearing liver lesions eventually being diagnosed as neoplastic lesions,^26^, ^27^, ^28^ including neuroendocrine tumor.^29^, ^30^

Neuroendocrine tumors (NET), traditionally referred to as carcinoid tumors, are neuroendocrine cell neoplasms originating from various organ systems such as the gastrointestinal tract, lung, pancreas, and genitor‐urinary organs. Pathologically they can also be assigned based on the mitotic activity in lesion, Ki—67 index and spectrum of differentiation from “low‐grade” well differentiated tumor(Grade 1) to “high‐grade” poorly differentiated carcinoma(Grade 3) with an aggressive biology and poorer prognosis. An epidemiology study compiling the NET cases from Norway and North America based on surveillance, epidemiology, and End Results (SEER) program as well as the Norwegian registry of cancer (NRC) was conducted. It reported that in the NRC cohort, the most common NET location was noted to be small intestine(26%), while in the SEER cohort, there was a high prevalence of bronchopulmonary NET(32%) in the white population and rectal NET (27%) in the black population.^31^ The overall incidence of neuroendocrine tumors was noted to be higher in men. The highest proportion of localized disease was noted in rectal and Meckel diverticulum NET corresponding to their favorable prognosis. Another study on identifying prognostic factors in patients with midgut neuroendocrine tumors with liver metastases showed a median survival of 7.69 years from the time of diagnosis and 5.95 years from the time of diagnosis of liver metastases.^32^ Further analysis reported that resection of liver metastases, resection of small bowel primary, treatment with somatostatin analogue therapy, and treatment with peptide receptor therapy were associated with improved prognosis.^32^

We chose to share this case report with the medical fraternity since such a presentation of a metastatic neuroendocrine tumor of ileum masquerading as liver abscesses has not been reported, and to acknowledge the rewards of ensuring beneficence toward our patients until an accurate diagnosis is made. Our patient with acute presentation of fever, abdominal pain, and transaminitis in the appropriate demographic context was evaluated using a stepwise process. Premature halting of the diagnostic would not have been appropriate since discerning infectious from malignant liver lesions radiologically can be challenging. This underscores the importance of following up on our patients both clinically and radiologically, especially when the diagnosis is uncertain and that repeating procedures such as biopsies may be needed. Our patient was able to connect with the appropriate level of care and surgical intervention occurred within 6 months. The patient was quite thankful for our team's persistence in pursuing his case for a diagnosis. This case also reinforces that the involvement of an Infectious Diseases consultant is associated with higher rates of accurate diagnosis and appropriate treatment.^33^

## CONFLICT OF INTEREST

None declared.

## AUTHOR CONTRIBUTIONS

AA: involved in data collection, analysis and interpretation, conceptualization and design, manuscript preparation, editing and review. MG: involved in data analysis and interpretation, report design, manuscript editing and review. JG: involved in data analysis and interpretation, report design, manuscript editing and review. DW: involved in data analysis and interpretation, report design, manuscript editing and review. This is to certify that all the authors have reviewed and contributed to the preparation of this manuscript and is in agreement with the final manuscript that we are submitting for the consideration of publication.
